# Complete Genome Sequence of Hypervirulent Streptococcus pyogenes *emm*3 Strain 1838

**DOI:** 10.1128/MRA.01494-18

**Published:** 2019-01-10

**Authors:** Benfang Lei, Anthony R. Flores, Carl Yeoman, Mengyao Liu

**Affiliations:** a Department of Microbiology and Immunology, Montana State University, Bozeman, Montana, USA; b Division of Infectious Diseases, Department of Pediatrics, Center for Antimicrobial Resistance and Microbial Genomics, McGovern Medical School, University of Texas Health Science Center at Houston, Houston, Texas, USA; c Department of Animal & Range Sciences, Montana State University, Bozeman, Montana, USA; University of Arizona

## Abstract

We report the complete genome sequence for Streptococcus pyogenes strain 1838 (type *emm*3) isolated from a patient with toxic shock syndrome. The strain lacked the *speK*- and *sla*-encoding prophage frequently encountered among *emm*3 strains and possessed an Arg66His mutation in CovR of the 2-component virulence regulatory system CovRS.

## ANNOUNCEMENT

Streptococcus pyogenes (group A *Streptococcus* [GAS]) strain 1838 was isolated from a patient with toxic shock syndrome in 2011 by the Streptococcus Laboratory at the Centers for Disease Control and Prevention (CDC) (1). The strain was included in a study to compare contemporary pharyngeal and invasive S. pyogenes isolates in their capacity to acquire *covRS* mutations using a mouse model of skin infection (2). CovRS (also known as CrsRS) is a 2-component regulatory system known to regulate expression of multiple GAS virulence factors (3–5). Naturally occurring CovRS mutations enhance expression of virulence genes and simultaneously downregulate the protease *speB*, which leads to hypervirulence (6–8). Among 6 SpeB-positive *emm*3 GAS strains, mice infected with strain 1838 demonstrated increased mortality in an *in vivo* subcutaneous infection assay for selection of CovRS mutants (2). Like *emm*3 strain MGAS315 (9), strain 1838 can invade the vascular system in a mouse model of pulmonary infection.

To understand the basis for hypervirulence, we sequenced the genome of strain 1838 using reads generated with both the PacBio RS II system and the Illumia MiSeq (300-bp, paired-end) instrument by the W.M. Keck Foundation Biotechnology Resource Laboratory at Yale University and Otogenetics Corporation, respectively. For DNA extraction, bacteria were streaked from a vial of the frozen isolate from the CDC on a Todd-Hewitt broth/2% yeast extract agar plate and incubated for 8 h at 37°C in 5% CO_2_. Genomic DNA from the bacteria was extracted using the MasterPure Gram-positive DNA purification kit (Lucigen/Epicentre, catalog number MGP04100) following the manufacturer’s protocol with the following modifications: bacteria from the plate were washed with 1-ml phosphate-buffered saline (PBS) 3 times and treated with 0.2-µg proteinase K in 1-ml PBS at 37°C for 2 h; the bacteria were pelleted by centrifugation, washed with 1-ml PBS 3 times, and resuspended in 150-µl Tris-EDTA buffer; and the bacterial suspension was mixed first with 10 µl of 1.0-mg/ml PlyC (10) and then with 150-µl lysis buffer and a 175-µl precipitation solution of the MasterPure kit. PacBio sequencing generated 132,929 reads with an average read length of 9,788 bp, and Illumina sequencing generated 14,839,914 reads and 1,869,829,164 bp. Hybrid assembly (i.e., long- and short-read sequences) using the SPAdes assembler (v 3.12.0) (11) and default parameters yielded a complete genome with greater than 1000× coverage. The complete genome was polished using Pilon (v 1.22) (12). The polished complete genome was annotated using the Prokaryotic Genome Annotation Pipeline at the National Center for Biotechnology Information (13).

In comparison with the genome of the *emm*3 strain MGAS315 (14), the strain 1838 genome shows a chromosomal inversion known to occur in approximately one-quarter of GAS genomes, presumably from a recombination event at homologous copies of *comX/sigX* (15). The inversion was identified using whole-genome alignments to completed serotype M3 GAS genomes with progressiveMauve (16). The inversion was verified by aligning both short and long reads to the completed 1838 genome and thus is unlikely to be a misassembly. Analysis of the strain 1838 genome with PHASTER (17) found 4 intact prophages, encoding *speC*, *speA*, and *spd1*. From the mid-1980s through in the early 2000s, virtually all *emm*3 strains had a 315.4-like prophage encoding *speK* and *sla* (14). However, like many *emm*3 strains from approximately 2007 to 2009 (18), strain 1838 lacked the *speK*- and *sla*-encoding prophage. Strain 1838 does have 315.3-, 315.5-, and 315.6-like prophages. Polymorphisms relative to MGAS315 were identified with NUCmer (v 3.1) (19) and by mapping Illumina short-read sequences from strain 1838 to MGAS315 with a custom pipeline as described by Long et al. (20). Excluding the prophage sequences, there were 189 single-nucleotide polymorphisms (SNPs) and 13 insertions/deletions (indels) in strain 1838 relative to MGAS315. Based on the chromosomal SNP data, strain 1838 appears to be closely related to *emm*3 strains that were responsible for a dramatic upsurge of M3 invasive infections in the United Kingdom in 2008 and 2009 (18) and consistent with currently circulating *emm*3 strains in the United States (21).

The 189 chromosomal SNPs between MGAS315 and strain 1838 contain 39 missense mutations, including one in each of *covR*, *covS*, and *ropB*. The *ropB* and *covS* SNPs are consistent with known polymorphisms in MGAS315 (8, 22), and the SNP in *covR* results in an arginine-to-histidine amino acid change at position 66 (Arg66His) in strain 1838 CovR. No other mutations were found in the known virulence genes and regulators. The Arg66His mutation is near the phosphorylation sites of CovR (aspartate at position 53, D53, and threonine at position 65, T65) (23), which may affect the phosphorylation of CovR and lead to enhanced virulence gene expression and hypervirulence of strain 1838.

### Data availability.

The genome sequence of GAS strain 1838 has been deposited in GenBank with the accession number CP029694. Raw sequences were deposited in the NCBI SRA database under BioProject number PRJNA473837.
